# Supplementary material for: Anterograde signaling controls plastid transcription via sigma factors separately from nuclear photosynthesis genes
Source: Nat Commun. 2022 Dec 2;13:7440. doi: 10.1038/s41467-022-35080-0 (PMC9718756; doi:10.1038/s41467-022-35080-0)
Supplement: Supplementary file 1 — Supplementary Information [file 41467_2022_35080_MOESM1_ESM.pdf]

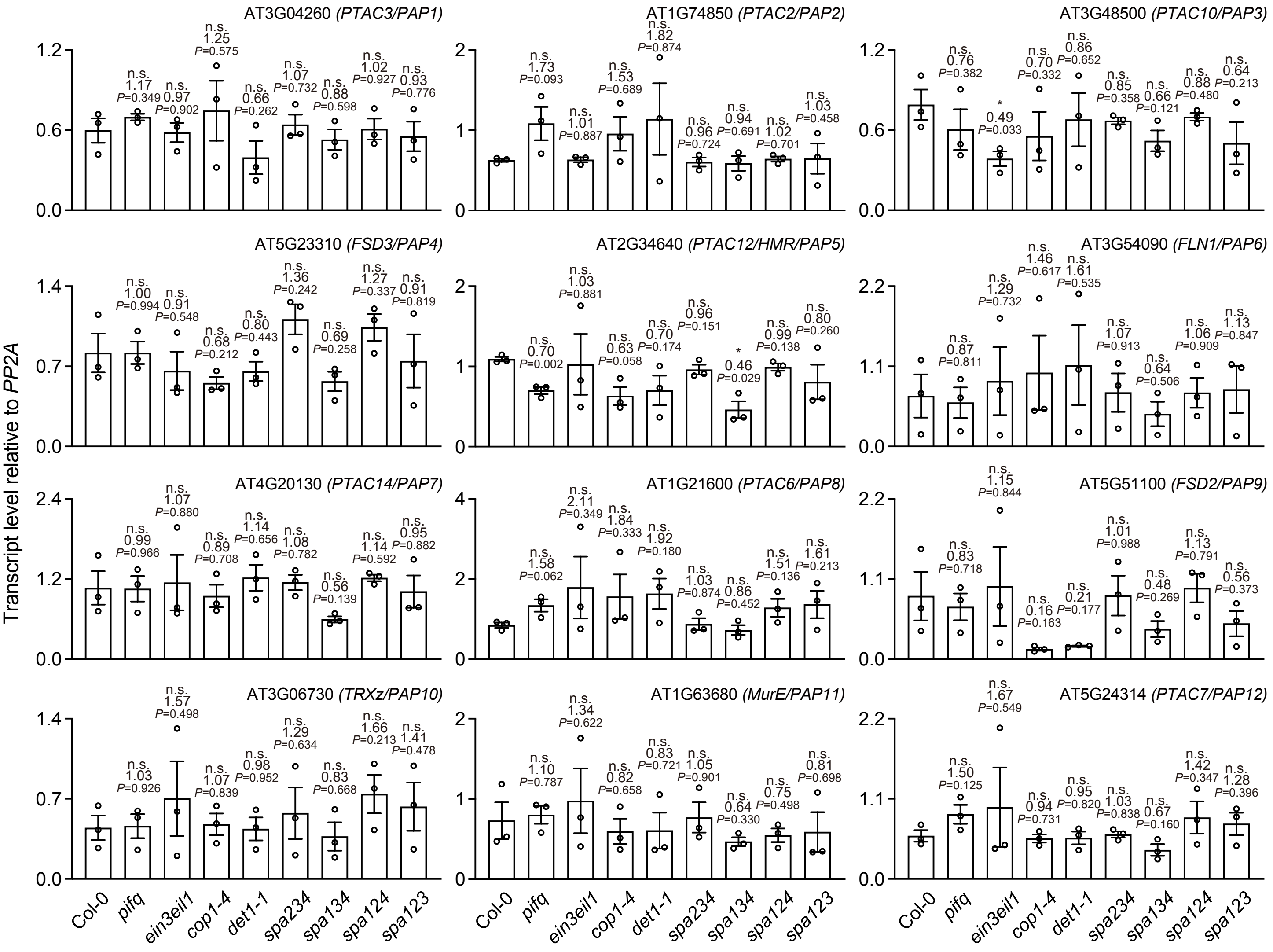

Supplementary Fig. 1. The transcript levels of *PAPs* were not altered in dark-grown de-etiolated mutants. qRT-PCR results showing the steady-state transcript levels of the 12 *PAP*s in 4-d-old dark-grown seedlings of Col-0, *pifq*, *ein3/eil1*, *cop1-4*, *det1-1*, *spa234*, *spa134*, *spa124*, and *spa123*. If the change between the mutant and Col-0 was less than twofold or not statistically significant based on two-tailed Student's t-test, it is labeled as n.s. (not significant). Error bars represent the s.e. of three biological replicates, and the centers of the error bars represent the mean values. The source data underlying the qRT-PCR analysis are provided in the Source Data file.

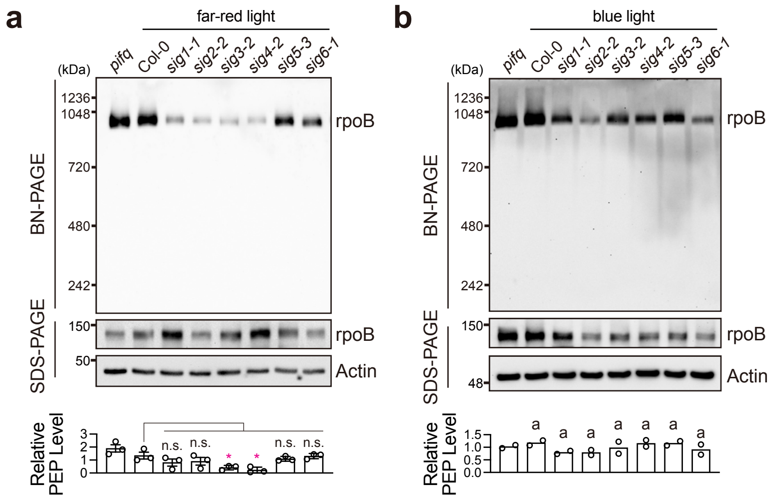

Supplementary Fig. 2. PEP assembly in *sig1-6* mutants in far-red and blue light. Immunoblots showing the levels of the PEP complex and the PEP component rpoB in 2-d-old Col-0 and *sig1-6* seedlings grown in 10 μmol m^{-2} s^{-1} monochromatic far-red (**a**) or blue (**b**) light. Total protein was isolated under either native or denaturing conditions and resolved via BN-PAGE or SDS-PAGE to assess the fraction of rpoB in the PEP complex or the amount of total rpoB by immunoblots, respectively. Dark-grown *pifq* seedlings were used as a control. Actin was used as a loading control. The graph below the immunoblots shows the relative PEP levels, which were estimated using the relative level of rpoB in the PEP complex (BN-PAGE) divided by the relative level of denatured rpoB (SDS-PAGE) in each sample. Asterisks in **a** indicate statistically significant differences compared with Col-0 based on two-tailed Student's t-test (* $P \leq 0.05$, n = 3 biological replicates). Different letters in **b** denote statistically significant differences in the relative PEP levels among the genotypes (ANOVA, Tukey's HSD, $P \leq 0.05$, n=2).

Supplementary Table 1. List of photosynthesis-associated nuclear genes (PhANGs) and plastid genes (PhAPGs) in *Arabidopsis*. LHCs, genes encoding light harvesting complex proteins; CHLs, genes encoding enzymes in chlorophyll biosynthesis; nPEACs, nuclear genes associated with photosystems, electron transport, ATP synthase, and the Calvin cycle.

Subcategories	Functional groups	PhANGs		PhAPGs	
LHCs	Light harvesting antenna	*Lhca1*	At3g54890		
		Lhca2	At3g61470		
		Lhca3	At1g61520		
		Lhca4	At3g47470		
		Lhca5	At1g45474		
		Lhca6	At1g19150		
		Lhcb1.1/CAB2	At1g29920		
		Lhcb1.2/CAB3	At1g29910		
		Lhcb1.3/CAB1	At1g29930		
		Lhcb1.4	At2g34430		
		Lhcb1.5	At2g34420		
		Lhcb2.1	At2g05100		
		Lhcb2.2	At2g05070		
		Lhcb2.3	At3g27690		
		Lhcb3	At5g54270		
		Lhcb4.1	At5g01530		
		Lhcb4.2	At3g08940		
		Lhcb4.3	At2g40100		
		Lhcb5	At4g10340		
		Lhcb6	At1g15820		
CHLs	Chlorophyll biosynthesis	*GTS*	At5g26710	*trnE*	AtCg00250
		HEMA1	At1g58290		
		HEMA2	At1g09940		
		HEMA3	At2g31250		
		GSA1	At5g63570		
		GSA2	At3g48730		
		FLU	At3g14110		
		ALAD1/HEMB1	At1g69740		
		ALAD2/HEMB2	At1g44318		
		PBGD	At5g08280		
		UROS	At2g26540		
		UROD1/HEME2	At2g40490		
		UROD2/HEME1	At3g14930		
		CPO1/HEMF1	At1g03475		
		CPO3/HEMN1	At5g63290		
		PPO1	At4g01690		
		PPO2	At5g14220		
		CHLI1	At4g18480		
		CHLI2	At5g45930		
		CHLD	At1g08520		
		CHLH/GUN5	At5g13630		
		CHLM	At4g25080		
		CHL27/CRD1	At3g56940		
		PORA	At5g54190		
		PORB	At4g27440		
		PORC	At1g03630		
		DVR	At5g18660		
		CHLG	At3g51820		
		CHLP	At1g74470		
		CAO	At1g44446		
		FC1	At5g26030		
		FC2	At2g30390		
		HO1/GUN2	At2g26670		
		HO2	At2g26550		
		HO3	At1g69720		
		HO4	At1g58300		
		HY2	At3g09150		
		UPM1	At5g40850		
		SIRB	At1g50170		
		ELIP1	At3g22840		
		ELIP2	At4g14690		
nPEACs	Phososystem II	*PsbO1*	At5g66570	*psbA*	AtCg00020
		PsbO2	At3g50820	*psbB*	AtCg00680
		PsbP1	At1g06680	*psbC*	AtCg00280
		PsbP2	At2g30790	*psbD*	AtCg00270
		PsbQ1	At4g21280	*psbE*	AtCg00580
		PsbQ2	At4g05180	*psbF*	AtCg00570
		PsbR1	At1g79040	*psbH*	AtCg00710
		PsbS	At1g44575	*psbI*	AtCg00080
		PsbTn	At3g21055	*psbJ*	AtCg00550
		PsbW	At2g30570	*psbK*	AtCg00070
		PsbX	At2g06520	*psbL*	AtCg00560
		PsbY	At1g67740	*psbM*	AtCg00220
		Psb27	At1g03600	*psbN*	AtCg00700
		Psb28	At4g28660	*psbTc*	AtCg00690
		Psb29	At2g20890	*psbZ*	AtCg00300
		Psb32	At1g54780		

**Photosystem I**	*PsaD1*<br>*PsaD2*<br>*PsaE1*<br>*PsaE2*<br>*PsaF*<br>*PsaG*<br>*PsaH1*<br>*PsaH2*<br>*PsaK*<br>*PsaL*<br>*PsaN*<br>*PsaO*<br>*PsaP*	At4g02770<br>At1g03130<br>At4g28750<br>At2g20260<br>At1g31330<br>At1g55670<br>At3g16140<br>At1g52230<br>At1g30380<br>At4g12800<br>At5g64040<br>At1g08380<br>At2g46820	*psaA*<br>*psaB*<br>*psaC*<br>*psaI*<br>*psaJ*	AtCg00350<br>AtCg00340<br>AtCg01060<br>AtCg00510<br>AtCg00630
**Cytochrome b_6f**	*PetC*<br>*PetM*	At4g03280<br>At2g26500	*petA*<br>*petB*<br>*petD*<br>*petG*<br>*petL*<br>*petN*	AtCg00540<br>AtCg00720<br>AtCg00730<br>AtCg00600<br>AtCg00590<br>AtCg00210
**Other electron transfer components**	*PC1/PetE1*<br>*PC2/PetE2*<br>*FD1/PetF2*<br>*FD2/PetF1*<br>*FNR1/PetH1*<br>*FNR2/PetH2*<br>*PetJ*	At1g76100<br>At1g20340<br>At1g10960<br>At1g60950<br>At5g66190<br>At1g20020<br>At5g45040		
**ATP synthase**	*AtpC1*<br>*AtpC2*<br>*AtpD*<br>*AtpG*	At4g04640<br>At1g15700<br>At4g09650<br>At4g32260	*atpA*<br>*atpB*<br>*atpE*<br>*atpF*<br>*atpH*<br>*atpI*	AtCg00120<br>AtCg00480<br>AtCg00470<br>AtCg00130<br>AtCg00140<br>AtCg00150
**NADH dehydrogenase**	*NdhL*<br>*NdhM*<br>*NdhN*<br>*NdhO*<br>*NdhS*<br>*NdhT*<br>*NdhU*<br>*NdhV*<br>*PnsB1*<br>*PnsB2*<br>*PnsB3*<br>*PnsB4*<br>*PnsB5*<br>*PnsL1*<br>*PnsL2*<br>*PnsL3*<br>*PnsL4*<br>*PnsL5*	At1g70760<br>At4g37925<br>At5g58260<br>At1g74880<br>At4g23890<br>At4g09350<br>At5g21430<br>At2g04039<br>At1g15980<br>At1g64770<br>At3g16250<br>At1g18730<br>At5g43750<br>At2g39470<br>At1g14150<br>At3g01440<br>At4g39710<br>At5g13120	*ndhA*<br>*ndhB.1*<br>*ndhB.2*<br>*ndhC*<br>*ndhD*<br>*ndhE*<br>*ndhF*<br>*ndhG*<br>*ndhH*<br>*ndhI*<br>*ndhJ*<br>*ndhK*	AtCg01100<br>AtCg00890<br>AtCg01250<br>AtCg00440<br>AtCg01050<br>AtCg01070<br>AtCg01010<br>AtCg01080<br>AtCg01110<br>AtCg01090<br>AtCg00420<br>AtCg00430
**Calvin cycle**	*RBCS1A*<br>*RBCS1B*<br>*RBCS2B*<br>*RBCS3B*<br>*PGK1*<br>*PGK2*<br>*CP12-1*<br>*CP12-2*<br>*CP12-3*<br>*GAPCP1*<br>*GAPCP2*<br>*GAPA-1*<br>*GAPA-2*<br>*GAPB*<br>*TIM*<br>*FBA1*<br>*FBA2*<br>*FBA3*<br>*FBP/HCEF1*<br>*RPE*<br>*SBPASE*<br>*TKL1*<br>*TKL2*<br>*PRK*<br>*RCA*<br>*CA1*<br>*CA2*<br>*CA5*	At1g67090<br>At5g38430<br>At5g38420<br>At5g38410<br>At3g12780<br>At1g56190<br>At2g47400<br>At3g62410<br>At1g76560<br>At1g79530<br>At1g16300<br>At3g26650<br>At1g12900<br>At1g42970<br>At2g21170<br>At2g21330<br>At4g38970<br>At2g01140<br>At3g54050<br>At5g61410<br>At3g55800<br>At3g60750<br>At2g45290<br>At1g32060<br>At2g39730<br>At3g01500<br>At5g14740<br>At4g33580	*rbcL*	AtCg00490

Supplementary Table 2. List of RNA-seq datasets used in this study.

BioProject	Genotype	Growth condition	Reference
PRJNA259643	*pifq, det1-1*	4-d-old, dark grown, 1X MS, 1% sucrose	Dong, J. *et al.* Arabidopsis DE-ETIOLATED1 represses photomorphogenesis by positively regulating phytochrome-interacting factors in the dark. *Plant Cell* **26**, 3630–3645 (2014).[29]
PRJNA480268	*ein3/eil1*	2.5~3-d-old, dark grown,<br>1X MS, 1% sucrose	Huang, P. *et al.* Integrated Regulation of Apical Hook Development by Transcriptional Coupling of EIN3/EIL1 and PIFs in *Arabidopsis*. *Plant Cell* **30***,* 1971-1988 (2018).[48]
PRJNA448672	*cop1-4, spaq*	3-d-old, dark grown, 1X MS,	Pham, V. N. *et al.* Molecular bases for the constitutive photomorphogenic phenotypes in Arabidopsis. *Development* **145**, (2018).[49]
PRJNA182723	*pifq*	2-d-old, dark grown	Pfeiffer, A. *et al*. Combinatorial complexity in a transcriptionally centered signaling hub in Arabidopsis. *Mol. Plant* **7**, 1598–1618 (2014).[50]
PRJNA610701	Col-0	3-d-old, dark-to-light transition	Kurihara, Y. *et al.* Time-course transcriptome study reveals mode of bZIP transcription factors on light exposure in *Arabidopsis*.", *Int J Mol Sci* **21**,1993 (2020).[58]

Supplementary Table 3. qRT-PCR primers for the nuclear genes examined in this study.

Accession	Gene name	Forward primer	Reverse primer
AT1G13320	*PP2A*	TATCGGATGACGATTCTTCGTGCAG	GCTTGGTCGACTATCGAATGAGAG
AT1G29920	*LHCB1.1*	GAGCCAAGTTCTATCTGTTTG	TCTACCATCCACCACAAACAC
AT2G34430	*LHCB1.4*	TCCTGCTTTGACCGGAAAGG	GGCTTTGCGCATGGTGATTC
AT3G54890	*LHCA1*	TCTGCTCCTGGTGACTTTGG	CCAGGAACAGCGAGCATAG
AT5G64040	*PsaN*	GCGAGAGCATTCACTGTTCA	TTCGCATTCCAAAGCAATATC
AT4G09650	*AtpD*	GAGGTTAGGTCGGTGGTGAA	AACCGTCTTAACCCGAACATT
AT1G12900	*GAPA-2*	GCACCTGGAAAAGGTGACAT	ACGAATGGAGCGAGACAGTT
AT1G64860	*SIG1*	AACTAAAACACGCAGCGAGGA	TCTTAAGGATCATTGCCTCCATTT
AT1G08540	*SIG2*	AGTCCAGAATGATAAGATTGCC	CTCTTTCACCCTATATGTTGCT
AT3G53920	*SIG3*	GTCTTTGTACATCCTCATCCT	TCTGCTTTCCTCTTTGACTG
AT5G13730	*SIG4*	CCATCTCCTTCTTTATCATCCC	CTATCAACCACTCTATCCACTG
AT5G24120	*SIG5*	GTGGTGAGGAGAAGAAAGTG	GCAATCCGTTTATCAAGACTC
AT2G36990	*SIG6*	CGGGCATTGTACAGGCTTAAG	AGCATGAAGTCCATGGCTGTTC
AT3G04260	*PAP1/pTAC3*	TCGCTGAGAAAGGAGCTAGG	GCGAGGATTTCTAGGCCTCT
AT1G74850	*PAP2/pTAC2*	TTGAACCGGACATGGAGACA	GGTGTTGAAAGCAACAAGCG
AT3G48500	*PAP3/pTAC10*	CATACCGGTTTCGGTTTCCC	CCTTCCTCCTCTGGCTTTGA
AT5G23310	*PAP4/FSD3*	CTGAACCACTTGGTGTCGTG	TTGGTTGGGACTTGGGACTT
AT2G34640	*PAP5/HMR*	CCAGTAATTGTATTGTGCAGAGAC	CACTTACATCACCATCTCCATC
AT3G54090	*PAP6/FLN1*	AGTCGCAAAGGGAGGAAGAA	TCGTCGTACGGAAACTCGAT
AT4G20130	*PAP7/pTAC14*	ATCGCTGCAGCAAGAACATT	TGCTCTGCTGTTGTGGGATA
AT1G21600	*PAP8/pTAC6*	AATGTCAAAGTCGCCGACAG	GTTCGGTGGTCACAGAATGG
AT5G51100	*PAP9/FSD2*	AGCCGGGAAACCTTGGATTA	CTCCCAGAAGAACTCGTGGT
AT3G06730	*PAP10/TrxZ*	GGAAGGTGCCGTTGATTGTT	GCGTGCAAACTCGTACTCAT
AT1G63680	*PAP11/MurE*	GCCGATGTTCACCCGTTAAA	AGAGGAGCTCCAACAGCAAT
AT5G24314	*PAP12/pTAC7*	AGCGTAGCAGCTGAAGCAAC	AAAGCCGAGATCGTTCACAC

Supplementary Table 4. Primers used for cDNA synthesis and qRT-PCR analysis of plastid genes.

Accession	Gene name	Primer for cDNA synthesis	Primer pair for qRT-PCR
ATCG00020	*psbA*	TAGATGGAGCCTCAACAGCAGCTA	ACATTTCTTCTTAGCGGCTT
			CGTCCTTGACTATCAACTACTGA
ATCG00680	*psbB*	CATCCAAATCTGGATCAATACCAG	GAATTAGATCGTGCGACTTTGA
			CTAGCACCATGCCAAATGTGTC
ATCG00490	*rbcL*	CTTCACAAGCAGCAGCTAGTTCAGG	GGAGATGATTCTGTACTACAAT
			GTCCCTCATTACGAGCTTGTAC
