# Supplementary material for: Anterograde signaling controls plastid transcription via sigma factors separately from nuclear photosynthesis genes
Source: Nat Commun. 2022 Dec 2;13:7440. doi: 10.1038/s41467-022-35080-0 (PMC9718756; doi:10.1038/s41467-022-35080-0)
Supplement: Supplementary file 3 — Description of Additional Supplementary Files [file 41467_2022_35080_MOESM3_ESM.pdf]

## Description of Additional Supplementary Files:

**Supplementary Dataset 1.** Transcriptomic analysis of genome-wide PhANGs in de-etiolated mutants. Relative transcript levels of the 149 PhANGs in dark-grown seedlings of pifq (4-d-old), det1-1 (4-d-old), ein3/eil1 (2.5-d old), cop1-4 (4-d-old), and spaq (3-d-old) are shown as fold-changes between the levels in the mutants and their respective Col-0 controls. Genes that are significantly upregulated in the mutants by at least twofold are labeled in magenta. The accession numbers of the RNA-seq data used in this study and their references are listed in Supplementary Table 2.

**Supplementary Dataset 2.** Transcriptomic analysis of genome-wide PhANGs in 2-d-old and 4- d-old dark-grown pifq seedlings. Relative transcript levels of the 149 PhANGs in dark-grown seedlings of 2-d-old pifq and 4-d-old pifq are shown as fold-changes between the levels in pifq and their respective Col-0 controls. Genes that are significantly upregulated in the mutants by at least twofold are labeled in magenta. The accession numbers of the RNA-seq data used in this study and their references are listed in Supplementary Table 2.
